# 肺癌术后胸腔闭式引流术应用的新观念

**DOI:** 10.3779/j.issn.1009-3419.2010.11.01

**Published:** 2010-11-20

**Authors:** 辉 时, 龙勇 梅, 国卫 车

**Affiliations:** 610041 成都，四川大学华西医院胸外科 Department of Toracic Surgery, West China Hospital, Sichuan University, Chengdu 610041, China

**Keywords:** 胸腔闭式引流术, 肺叶切除术, 肺肿瘤, Closed chest drainage, Lung lobectomy, Lung neoplams

## Abstract

肺叶切除术后应用传统胸腔闭式引流术，被大多数胸外科医生所采用的主要原因：一是引流效果好；二是经验、习惯和观念。外科技术的发展和医疗观念的更新使传统胸腔闭式引流术在临床应用中的不足越来越明显，但尚未引起足够重视。近几年对肺癌术后引流问题无论是应用方法还是观念都有更新和发展。本文将结合国内外研究进展和我们工作中的体会，从以下三方面进行概述：一是胸腔引流术应用现状和存在问题；二是常规水封引流系统加用负压吸引之优势与不足；三是单胸腔引流管的临床应用进展与争议。

## Abstract (en)

It is widely accepted by most thoracic surgeons that a complete control on fluid and air in the pleural space could only be maintained by conventional closed chest drainage (two chest tubes). With the development of medical models and devices in pneumonectomy, the validity of this concept is being questioned. In this review, we summarized that: the current situation and problems of closed chest drainage; the advantage and disadvantage of water seal with or without suction on chest drainage; and the advances and contradiction of single chest tube drainage after pneumonectomy.

胸腔闭式引流术经过近百年的临床应用和不断改进，已成为被广泛接受和应用的方法，且被教科书和各种临床指南奉为经典。肺癌术后应用传统胸腔引流术（双引流管）能够充分排除胸腔气体和液体并促使肺复张，是其被胸外科医生广泛接受和应用的主要原因。近30年来，尽管肺癌外科技术和方法不断发展和更新，而大多数胸外科医生对应用已久且习惯的引流方式不愿更改，情有独钟。当前人们更加关注肺癌患者的快速康复和生活质量的提高，使传统引流方法的缺点和不足呈现得越来越明显，主要表现在：术后疼痛，既不利于患者排痰和肺复张，又不利于患者活动和物理康复训练等。因此，近年来探索胸腔引流的新方法和传统引流方法的改进也成为研究的热点。本文对目前关于肺癌术后胸腔闭式引流和新进展概述如下。

## 1 肺叶切除术后胸腔闭式引流术的应用现状和存在问题

肺叶切除术后应用单水封瓶胸腔闭式引流系统处理积液、积气，此方法的最大优点是方便简单、成本低和效果好，奠定了现代肺叶切除术后胸腔闭式引流系统的开端^[1]^。Howe等^[2]^应用的三瓶胸腔闭式引流系统（收集瓶、水封瓶、压力瓶）是历史上沿用最久也是如今所有正在应用的胸腔闭式引流系统的发展基础（图 1）。截至1975年，由于没有统一生产和使用标准，无论是引流管/瓶的材料和大小，都是依据生产商或医生个人的喜好进行生产和提供，从而给临床胸腔引流系统管理和应用带来困难，且增加了相关并发症。1975年对300名胸外科医生进行胸腔闭式引流系统的问卷调查表明：82%医生应用多瓶（2个或3个）引流系统，28%用单瓶引流系统；59%用塑料瓶作为引流瓶，37%用玻璃瓶，4%用自制引流系统^[3]^。1998年问卷调查表明80%西班牙胸外科医生应用传统的胸腔闭式引流系统，且无改变的意愿^[4]^。2006年欧州胸外科医师协会对120个医疗中心的所有胸外科医生进行网上问卷调查，目的有三：一是当前胸腔闭式引流现状；二是未来技术发展方向；三是为胸腔闭式引流应用指南制订作准备。结果表明：55.9%的医生应用PVC硬管引流，38.4%应用硅橡胶管引流；43.4%应用水封瓶引流系统，其中单瓶引流占24.8%，双瓶18.2%；全肺切除术后，51.2%应用稳压引流系统，9%应用定期穿刺引流，39.8应用其它方法；87.8%医生偏爱加用负压吸引，其中77.7%医生认为加用负压吸引的指征为有持续性漏气^[5]^。结合实际，在我国大多数胸外科医生仍采用传统的胸腔引流方法，其应用和欧洲胸外科医师协会得出的结果相似。

**1:**
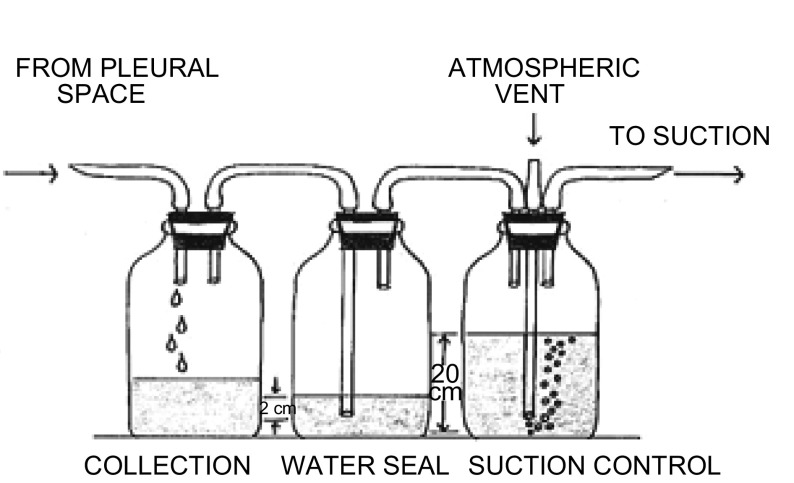
三瓶引流系统 Three-bottle drainage system

总之，从胸腔引流系统开始到现在的近百年间，其应用发展大概经历了以下几个阶段：第一个50年（1875年-1926年）确立了胸腔闭式引流系统基本方法和原则；第二个50年（1926年-1975年）奠定现代胸腔闭式引流系统的雏形，并在引流管管径大小、材料选择、引流瓶的大小、材料应用等方面进行了充分的研究和探索，基本达成共识；1975年-2000年间发表的主要关于胸腔闭式引流的文献集中讨论了胸腔引流系统的管理、并发症及其处理方法；从2000年至今，一些胸外科医生开始探讨和应用新的引流方法（如：单管引流、可弯曲的细孔径的引流管、不加用负压吸引），并与传统胸腔闭式引流进行随机对照研究。这些应用方法基本上克服了传统引流方法的缺点，而且引流效果优于或相当于经典的引流方法，并在一些单位开始应用。目前，大多数胸外科医生在肺癌切除术后仍应用传统的胸腔闭式引流作为主要引流方法，新的方法还未得到普遍接受。多数医生仍在根据个人或老师、单位的经验进行应用，既无标准又缺乏循证医学依据。

## 2 水封引流系统加与不加负压吸引应用的现状、优势和不足

胸腔闭式引流应用水封瓶作为密闭系统，将引流管置于水封瓶内水下2 cm-3 cm，这主要是利用重力作用维持胸膜腔内生理性负压和利于引流。在胸腔闭式引流术应用之初，为了达到尽快排气和排液，促进肺复张的目的，即有医生在水封引流基础上加用真空吸引泵或负压系统，这种方法在其早期就被大多数胸外科医生接受，并在长期临床实践中得出最理想负压为-20 cm H_2_O。但是最近临床随机对照研究结果表明，单纯水封瓶引流加不加负压吸引，对肺癌术后漏气时间、引流管持续时间、引流量、胸腔内积气、积液量、拔管时间、住院时间影响不大，可能还有负作用。Alphonso等^[6]^将239例行肺叶切除或楔形切除的患者随机分为两组[单纯水封瓶引流组和加用负压（-20 cm H_2_O）吸引组]，术后肺漏气 > 6 d的发生率在不加与加负压吸引组间无差异（7.8% *vs* 10.1%）（*P* > 0.05）；两组术后肺漏气引流时间也无差异。因此，作者推荐采用单纯的水封引流。Brunelli等^[7]^将肺叶切除术后第1天发生漏气的患者随机分为两组，一组单纯水封引流，另一组水封加用负压-20 cm H_2_O持续吸引：两组漏气时间分别为6.5 d *vs* 6.3 d，漏气延长发生率分别为27.8% *vs* 30.1%，两者均无统计学差异（*P* > 0.05）。Ayed等^[8]^将100例连续收治的行胸腔镜手术的自发性气胸患者术后分为水封引流加用和不加用负压吸引组，结果发现：负压吸引组中14%的患者漏气时间延长，而单纯引流组只有2%，单纯引流组和加用负压引流组的引流时间（2.7 d *vs* 3.8 d）和住院时间（3.8 d *vs* 4.8 d）均有统计学差异（*P* < 0.05）。Marshall等^[9]^将行肺叶切除术或楔形切除术的68例患者在术后进行短暂的负压吸引后，随机分为水封引流组和水封加用负压吸引组，结果单纯引流组的漏气时间和拔管时间（0.08 d和0.17 d）均显著短于加用负压吸引组（0.17 d和0.32 d）。Antanavicius等^[10]^回顾性分析了109例肺叶切除患者术后（分别应用水封引流加用和不加用负压吸引）的引流时间和住院时间，发现二者在单纯水封瓶引流组显著短于加用负压吸引组。Prokakis等^[11]^将肺叶切除的91例患者随机分为两组，水封引流组47例，加用负压（-15 cm H_2_O–-20 cm H_2_O）44例，结果表明两组的引流时间、死亡率、并发症发生率、引流效果、术后住院时间均无统计学差异（*P* > 0.05）。可见肺叶切除术后常规应用负压吸引是不必要的。但对持续漏气的患者，若肺已复张，持续负压吸引也不必要，而肺复张不好的患者应用负压吸引有助于肺复张和缩短漏气时间。Sanni等^[12]^对已发表文献分析发现，肺叶切除术后加用负压吸引对胸片显示大量气胸和大量漏气的患者而言，优于单纯引流。Okamoto等^[13]^回顾性分析了120例肺癌行肺叶切除术后发生漏气的患者应用水封加用负压吸引（-10 cm H_2_O）、47例肺癌肺叶切除术后发生漏气的患者应用水封引流作为对照组的效果，发现水封引流加用和不加用负压吸引对术后漏气时间和住院时间影响不大。Brunelli等^[14]^将肺癌行肺叶切除术后第1天发生漏气的94例患者随机分为单纯水封引流组和交替负压（-10 cm H_2_O）吸引组（晚上负压吸引，白天停用）。结果表明：交替负压吸引组的漏气时间、引流管留置时间、术后住院时间均显著短于单纯引流组。这种只在晚上用负压吸引的方式有利于患者白天的活动和康复治疗。

## 3 单胸腔引流管应用的优势与不足

肺叶切除术后传统的胸腔引流方法是两根引流管（一根在前上引流气体，另一根在后下引流液体）。肺叶切除术后（尤其是上叶）用单根引流管能否将气体和液体同时引流彻底，是否有助于肺复张，是否能达到双管引流的效果？从理论上讲，双管引流的效果应该优于单管引流；而单管引流的优势在于术后疼痛小、有利于患者活动和物理理疗训练、有助于痰液排出等。近年几项随机对照临床研究回答了这些问题，现分述如下：一是引流管选择（材料和孔径大小）；二是引流管放置方法和是否加用侧孔和负压吸引；三是引流效果是否和双引流管相当；四是单胸腔引流管临床应用的共识和争议。

### 3.1 引流管的材料和孔径大小

目前，世界范围内商家提供和临床应用的引流管的主要材料类型有：①聚氯乙烯材料（polyvinylchlorid, PVC），有硬直和半硬可弯曲两种，其中以硬直管应用较多；②硅橡胶管；③橡胶管等。以PVC材料应用为最多，其次为硅橡胶管，橡胶管应用已很少，但有的单位应用24号的橡胶蕈形尿管作为上胸腔引流管。最初应用时引流管径大小6F-40F不等。目前临床应用达成的共识是：28F、32F和36F应用于成人；而16F、20F和24F用于儿童^[1]^。橡胶引流管可曲性好，但容易折叠而导致引流不通畅，引流效果差，但引起疼痛轻；硬直PVC半透明引流管引流效果好且易观察，但引起疼痛重；硅橡胶管引流效果好，但不透明，不易观察。

### 3.2 引流管放置方法及是否需负压吸引和侧孔

由于单管胸腔引流尚处于探索阶段，尚无统一的标准。据报道的主要应用方法有：①32F聚乙烯管置入术侧切口下方肋间腋中线上，胸腔内经胸顶，且于引流管在胸腔内最低位另开孔，术后第1天间断负压（-15 cm H_2_O–-20 cm H_2_O）吸引^[15]^或持续负压（-20 cm H_2_O）吸引^[16]^；②19F BD硅橡胶管[Blake^®^ drain (BD)]从切口下方前胸壁进入胸腔内、向上绕过肺尖、向下直达胸腔背侧肋膈角，简称前后位（anterior-to-posterior, AP）（图 2A）。前后位术后一种是加用负压（-10 cm H_2_O）吸引，另一种是水封引流。另外，19F BD硅橡胶管从切口下方肋间腋中线上进入胸腔，经背侧向上经肺尖到前方向下到前胸腔最低位，为后前位（posterior-to-anterior, PA），常规应用-10 cm H_2_O负压吸引（图 2B）。据作者临床研究^[17]^提示：前后位加用负压吸引引流效果优于其它两种方法；③Argyle^©^28F（Sherwood Services AG, TYCO Healthcare, Ireland）引流管从切口下方进入胸腔从肋膈角指向胸腔中部，持续负压（-15 mmHg–-20 mmHg）吸引^[18]^；④19F BD从第2或第3肋间腋前线穿刺点进入胸腔，经胸顶部下降入背面的肋膈角^[19]^（图 3）；⑤单根可弯曲的24F BD通过切口下方肋间切开0.5 cm切口置入胸腔内，若是下叶切除则胸腔内引流管位置在胸腔后部靠近脊柱旁；若是上叶切除切口位置同前，只是将引流管中部先弯曲，则引流管的位置在胸腔内是自下向上，胸顶弯曲后向前下到肋膈角。两者均需持续负压（-20 cm H_2_O）吸引^[20]^。我们在临床上应用单根28F BD引流管，肺叶（不论是上、下叶）切除术后均从术侧腋中线第7肋间置入直达胸顶，不用加用负压吸引，但要注意体位引流（平躺或头低位），发现术后引流效果与双腔管相当。而术后疼痛却明显减轻，且有助于患者活动。

**2:**
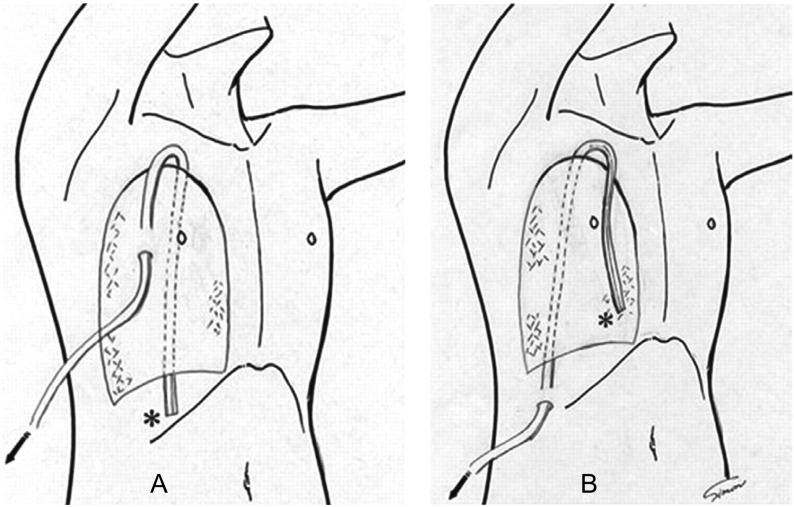
单胸腔管引流放置方法。A：前后位；B：后前位。 The method of placing a drain placement. A: anterior-toposterior, AP; B: posterior-to-anterior, PA.

**3:**
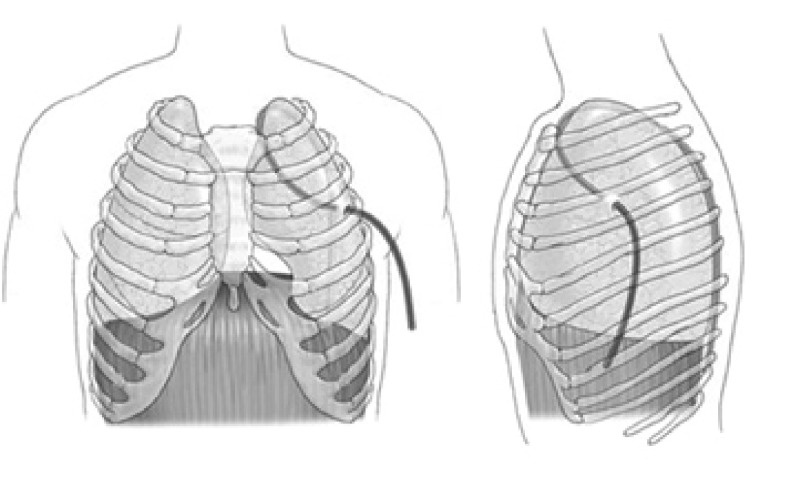
19F BD引流方法 Method of insertion of Blake drains

### 3.3 单根引流管的引流效果是否和双引流管相当

已有关于单引流管与传统双引流管的引流效果及相关并发症分析，均提示单管引流效果相当或优于双引流管，且并未增加相关并发症。Gómez-Carot等^[18]^应用24F BD单管引流60例与双管引流的59例随机对照研究表明，两种方法在术后的并发症和死亡率、皮下气肿、气液残留、拔管时间和需要再次置管方面均无统计学差异；而麻醉药用量则双管要多于单管，即单管引流引起疼痛轻。Icard等^[20]^应用24F BD对连续100例肺叶切除术后患者进行胸腔引流表明，无一例患者需要再次手术或置管，引流留置时间平均为5 d，90%患者能按时拔管，作者认为肺叶切除术后单管引流的有效性和安全性与双腔引流相当。Nakamura等^[19]^对134例肺叶切除患者分别应用19F BD和24F BD进行术后引流，发现术后0 h-12 h内28F引流管引流量显著多于19F，而19F则在12 h-24 h内显著多于28F，但是二者的引流总量无差异，且19F引流管口愈合明显优于28F。

但也有报道认为单胸腔引流管不但可使疼痛明显减轻，而且引流量也明显减少。Okur等^[15]^将100例肺叶切除术后应用32F BD引流的患者随机分为两组，结果表明：单管引流量[（600±43.2）mL]明显少于双管引流[（896±56.2）mL]（*P* < 0.001）；平均术后疼痛指数（visual analogue scale, VAS）在术后第2天单管（4.28±0.21）显著小于双管引流（5.10±0.23）（*P*=0.014）；而第2周VAS评分单管（1.48±0.13）明显好于双管（2.00±0.17）（*P*=0.01）。Pawelczyk等^[16]^对183例肺叶切除术后患者，93例应用单管引流，90例应用双管，结果显示，两组在引流量、需要支气管镜吸痰和再次置管、术后相关并发症及死亡率方面均无统计学差异；单管引流患者住院时间显著短于双管（7.6 d *vs* 9.0 d; *P*=0.001）；止痛药应用时间单管短于双管(4.8 d *vs* 5.6 d; *P*=0.000 1)；抗炎药应用时间单管短于双管（6.8 d *vs* 7.7 d; *P*=0.002）；术后第4天双引流管导致疼痛明显强于单管。单管引流每个患者治疗费用平均降低125欧元。Alex等^[21]^对120例肺癌肺叶切除术后引流分别应用单根28F BD和双引流管，每组各60例。结果发现单管与双管的住院时间（7.7 d *vs* 7.8 d; *P*=NS）、引流量（平均667 mL *vs* 804 mL；*P*=NS）、引流时间（平均4 d *vs* 4.3 d；*P*=NS）、镇痛时间（平均3.7 d *vs* 4.2 d；*P*=NS）均无统计学差异，但双管VSA评分高于单管（平均1.4 *vs* 1.02；*P*=0.02）。单管引流患者的平均住院费用比双管引流患者少3 300美元。

### 3.4 单胸腔引流管临床应用的共识和争议

肺叶切除术后应用胸腔闭式引流已成为一种规范，尽管很重要但过去30年却只有30篇文章发表，而实际上大多数心胸外科中心都在应用单管引流，只是没有当作可以推广的经验加以总结。然而大家目前对单管胸腔引流所达成的共识有：①单胸腔引流管临床应用的优势包括：疼痛减轻、利于患者术后活动和物理康复训练、引流管刺激胸膜引起的胸水减少、患者舒适度和依从性增加、利于皮肤切口愈合等；②引流管应用材料以PVC为主，孔径从19F-32F，28F应用最多，多数主张置于胸顶且于低位开侧孔，加用负压（-10 cm H_2_O–-20 cm H_2_O）间断或持续吸引；③若病例选择恰当，其引流效果优于或相当于传统双管引流，且不增加并发症或死亡率。现在争议最多的是对肺叶切除术后单管引流的适应症的掌握，普遍认为应根据术中情况，若估计术后漏气或胸水在可估计水平，可以应用单管引流。若有下列情况则应选择双管引流：①肺叶间裂发育不全；②术前接受放疗；③胸腔广泛粘连；④余肺正压通气后不能充满胸腔；⑤肿瘤侵犯胸壁（T_3_）；⑥术前有凝血障碍、心、肾、肝疾病^[22, 23]^。

## 4 问题与展望

肺叶切除术胸腔引流技术应用及发展近百年，随着医学模式转变与外科技术提高，一些传统、习惯的观念需要更新。基于经验的方法更应该通过系统和规范的研究方法进行评价。目前各种胸腔闭式引流方法的临床评价大多是通过各个单位或个别医生的经验，缺乏有力的循证医学证据，因此在临床应用中出现了各种各样的问题。主要有：①引流管的材料和孔径大小选择是基于商家和医院提供的引流管；②应用方法来自本单位和上级医生经验；③引流时间和拔管标准也无统一标准，主要根据指南或个人经验；④是否应用负压吸引更没有可参考的临床指南；⑤应用单引流管或双引流管的临床适应症难以确定。但是正如一些专家所说，过去30年大家都没有对胸腔引流术的应用足够的重视，但可喜的是现在一些专家已经或正在进行引流方法的改进，并应用可信度高的随机对照研究，且已取得了一些研究成果，如单引流管的应用，负压吸引等。因此，随着研究的进一步深入，胸腔引流技术的应用会更加规范，也有助于患者术后快速康复和提高生活质量。
